# In-line phase-contrast and grating-based phase-contrast synchrotron imaging study of brain micrometastasis of breast cancer

**DOI:** 10.1038/srep09418

**Published:** 2015-03-30

**Authors:** Sheng Huang, Binquan Kou, Yayun Chi, Yan Xi, Yixin Cao, Wenli Cui, Xin Hu, Zhimin Shao, Han Guo, Yanan Fu, Tiqiao Xiao, Jianqi Sun, Jun Zhao, Yujie Wang, Jiong Wu

**Affiliations:** 1 Department of Breast Surgery, Breast Cancer Institute, Shanghai Cancer Center, Department of Oncology, Shanghai Medical College, Fudan University, Shanghai, People's Republic of China; 2 Department of Physics and Astronomy, Shanghai Jiao Tong University, Shanghai, People's Republic of China; 3 School of Biomedical Engineering, Shanghai Jiao Tong University, Shanghai, People's Republic of China; 4 Med-X Research Institute, Shanghai Jiao Tong University, Shanghai, People's Republic of China; 5 Department of Pathology, Fudan University, Shanghai Cancer Center, Shanghai, People's Republic of China; 6 Department of Pathology, First Affiliated Hospital Xinjiang Medical University, Urumqi, Xinjiang Uygur Autonomous Region, People's Republic of China; 7 Shanghai Institute of Applied Physics, Chinese Academy of Sciences, Shanghai, People's Republic of China

## Abstract

Current bio-medical imaging researches aim to detect brain micrometastasis in early stage for its increasing incidence and high mortality rates. Synchrotron phase-contrast imaging techniques, such as in-line phase-contrast (IPC) and grating-based phase-contrast (GPC) imaging, could provide a high spatial and density imaging study of biological specimens' 3D structures. In this study, we demonstrated the detection efficiencies of these two imaging tools on breast cancer micrometastasis in an *ex vivo* mouse brain. We found that both IPC and GPC can differentiate abnormal brain structures induced by micrometastasis from the surrounding normal tissues. We also found that GPC was more sensitive in detecting the small metastasis as compared to IPC.

A renewed focus on the problem of metastasis is now obvious since it is responsible for about 90% of deaths from solid tumors^1^. The situation is similar for breast cancer in which most deaths are not due to the primary tumor, but to the metastases that occur in other organs of the body^2^. Approximately 10–15% of patients with breast cancer develop brain metastasis (BM) based on clinical evidence, these figures underestimate the true incidence rate since autopsy studies have shown an incidence rate up to 35%^3^,^4^. Recently, the clinical BM incidence rate is increasing owing to two primary reasons. The first one is that improved imaging techniques can detect smaller tumors than before. The second one is that improved local control and therapy for metastasis at the visceral organs can lead to prolonged survival^5^,^6^. As a result, the morbidity and mortality rates due to late-diagnosed BM are naturally projected to rise^7^. Currently, there is no consensus on how to screen for intracranial metastasis in patients with initial stage systemic cancers.

The detection of breast cancer metastasis at an early stage is critical for clinicians and patients to manage and predict breast cancer progression. Imaging is essential for the detection and diagnosis of BM. When patients present new neurological signs and symptoms, computerized tomography (CT) is the primary imaging modality because it can be easily performed with high adaptability, which is crucial for rapidly excluding life-threatening emergencies at low cost^8^,^9^. The current clinical imaging gold standard for detecting intracranial neoplasm is magnetic resonance imaging (MRI). With and without contrast agents, standard T1- and T2-weighted images provide excellent anatomic details and are highly sensitive in detecting the size and location of brain tumors, as well as identifying secondary changes, such as edema, hemorrhage, necrosis, mass effect, and signs of increased intracranial pressure. Advanced MRI techniques, such as magnetic resonance spectroscopy (MRS), magnetic resonance perfusion (MRP), diffusion weighted imaging (DWI), and diffusion tensor imaging (DTI), may also be used to help distinguish brain metastases from other pathologies and monitor treatment responses^10^. For lesions less than 5 mm in diameter, contrast-enhanced MRI can detect two- to three-folds more lesions than CT^9^,^10^. Other technique like 18-fluorodeoxyglucose positron emission tomography (FDG-PET) is important for staging in other areas of the body, but not as sensitive as MRI for evaluating BM^11^,^12^. The lower sensitivity is because the cerebral cortex is highly receptive to FDG^10^, which makes it difficult to discriminate the hypermetabolic metastases.

However, all these existing methods cannot detect or discriminate micrometastasis. Therefore, another method is required for early metastasis detection. Phase-contrast X-ray imaging techniques that use phase information, which correspond to the real part of the sample refraction index δ, provide better contrasts for light element samples^13^. Therefore, X-ray phase-contrast imaging techniques can significantly improve the image quality of soft tissue samples. Among the phase-contrast X-ray imaging techniques, the GPC imaging method was recently developed^14^,^15^ and has been widely applied for bio-medical imaging applications^16^,^17^,^18^,^19^. The imaging contrast with the GPC method is proportional to the first derivative of the real part of the samples' refraction index, which is very suitable for samples with smooth density variations. Three-dimensional quantitative information is also available from a GPC-CT^14^. The IPC imaging method also exploits the phase information from the sample, and its imaging contrast is proportional to the second derivative of the real part of the refractive index δ, which is more sensitive for sharp edge detections, such as for the imaging of blood vessel structures^20^.

In the present paper, we investigated the detection efficiencies of the IPC-CT and GPC-CT on micrometastasis in a mice brain metastasis model of human breast cancer. We designed this *ex vivo* study in order to evaluate the potential for future *in vivo* applications.

## Results

**Figure 1:**
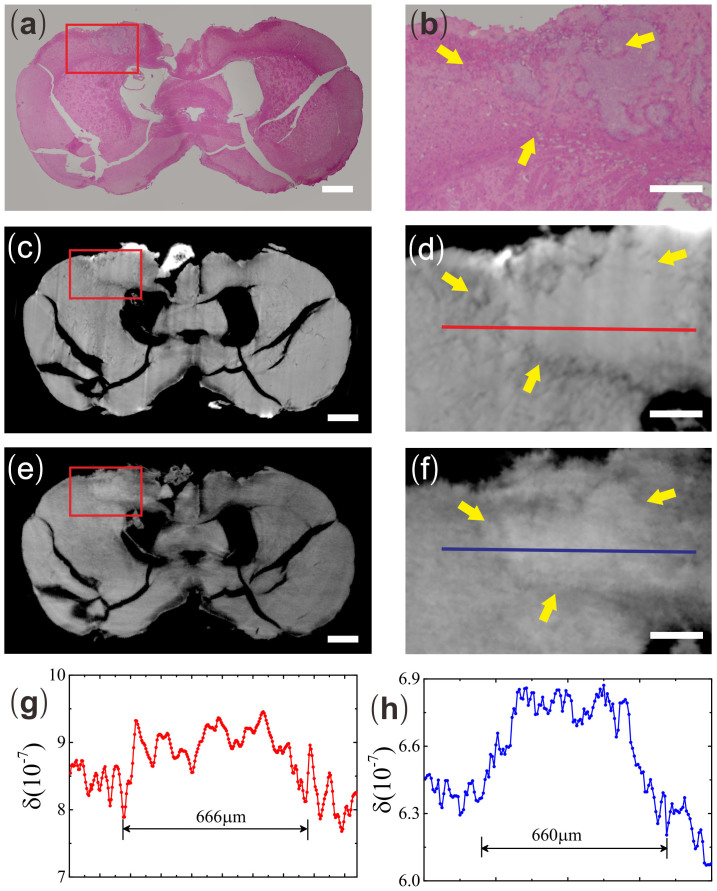
The brain slice and reconstructed tomogram in three formats, including IPC-CT (c) and GPC-CT (e), as well as H&E pathological graphs (a) in motor cortex metastasis. Enlargement of the motor cortex metastasis in (b) H&E pathological graph, (d) IPC-CT image and (f) GPC-CT (×4.3, 1170 × 790 μm^2^). (g) The δ values along the red line in Fig. 1d. (h) The δ values along the blue line in Fig. 1f. Scale bar: 500 μm (a, c, e) and 200 μm (b, d, f).

**Figure 2:**
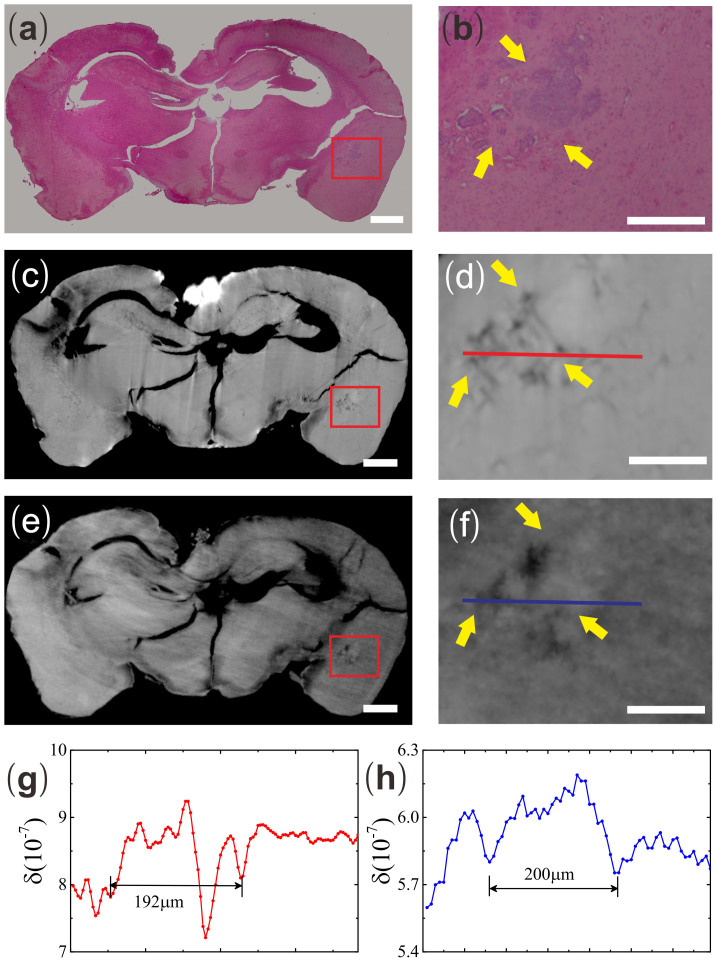
The brain slice and reconstructed tomogram in three formats, including IPC-CT (c) and GPC-CT (e), as well as H&E pathological graphs (a) in hippocampus metastasis. Enlargement of the hippocampus metastasis in (b) H&E pathological graph, (d) IPC-CT image and (f) GPC-CT image (×5.7, 700 × 580 μm^2^). (g) The δ values along the red line in Fig. 2d. (h) The δ values along the blue line in Fig. 2f. Scale bar: 500 μm (a, c, e) and 200 μm (b, d, f).

To evaluate the efficiencies of both IPC-CT and GPC-CT in the detection of breast cancer metastasis, the gold standard pathology was exploited. To avoid information loss, the sample was serially sectioned and stained with H&E (see Fig. 1(a) and 2(a)). The two abnormal structures identified by both IPC-CT (see Fig. 1(c) and 2(c)) and GPC-CT (see Fig. 1(e) and 2(e)) were consistent with metastases in the motor cortex and the hippocampus areas from H&E.

Analyzing δ of the micrometastasis and its peripheral tissue in motor cortex (see Fig. 1(d) and 1(f)), we found that in both IPC and GPC, δ values at the micrometastasis are higher than their peripheral tissues. And the width of the high δ value range from the line profiles taken across the micrometastasis suggests the size of the metastasis is on the order of several hundred microns. It is approximately 666 μm in IPC and 660 μm in GPC (see Fig. 1(g) and 1(h)).

In our experiment, IPC and GPC could easily differentiate motor cortex micrometastasis from the surrounding normal tissues based on the δ value. But for micrometastasis in the hippocampus whose width was approximately 200 μm, it was difficult to be identified by IPC unambiguously due to weak contrast. Instead, we first identified the micrometastasis in GPC image (see Fig. 2(e)). Then after it was confirmed by H&E (see Fig. 2(a)), we reanalyzed the IPC image at the same location (see Fig. 2(c)). The results are similar to those from the metastasis in motor cortex. By analyzing the line profiles taken across the hippocampus metastasis, we found the δ value at the metastasis by GPC is significantly higher than the peripheral normal tissues while the corresponding δ value at the metastasis by IPC was difficult to be distinguished from the background (Fig. 1(g) and 2(g)).

**Figure 3:**
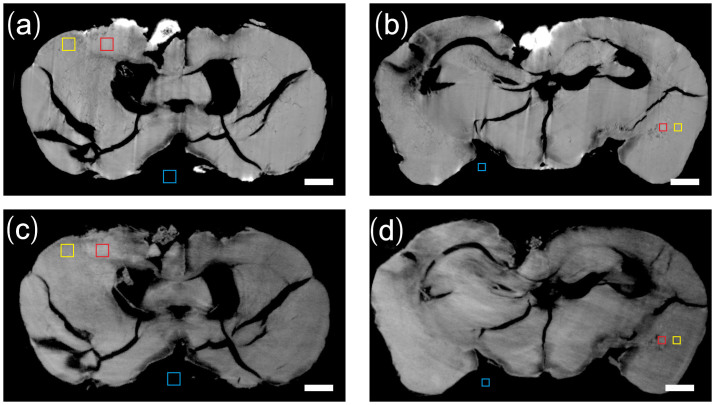
Three regions selected in IPC-CT image (a, b) and GPC-CT iamge (c, d) for CNR analysis. (1) red square: tumor (2) yellow square: surrounding region of the tumor (3) blue square: background region. The areas of the square regions in (a), (c) are 200 × 200 μm^2^ while 120 × 120 μm^2^ in (b), (d). Scale bar: 500 μm.

In order to quantify the visibility difference of the metastases by two techniques, we performed a CNR (Contrast-to-noise ratio) analysis in the selected homogeneous ROIs (region of interest) in each slice. We chose three regions including 1) the tumor (red square), 2) the surrounding region of the tumor (yellow square) and 3) the background region (blue square) in Fig. 3. ROIs with an areas of 200 × 200 μm^2^ were chosen for motor cortex micrometastasis and ROIs with areas of 120 × 120 μm^2^ for hippocampus micrometastasis. The CNR is calculated as follows:
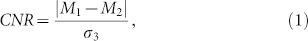
where *M*_1_ and *M*_2_ are the mean δ value of region (1) and (2), *σ*_3_ is the standard deviation of δ value in background region (3) which provides a measure of the image noise level. The uncertainty of the CNR was determined by standard error propagation method^21^. Table 1 lists the CNR of the ROIs in IPC and GPC. The CNR of GPC in motor cortex micrometastasis was 22.3% lower than that of IPC, while in the smaller hippocampus micrometastasis, the CNR of GPC was 267% higher than that of IPC.

The spatial resolutions of H&E sections, reconstructed IPC and GPC slices were obtained by Fourier analysis^22^,^23^. We calculated the radial spectral power (RSP) of region of interest (200 × 200 pixels) which is scaled by the RSP of a background region of same size. Similar to previous study, the spatial frequencies which have twice of the RSP value of the baselines in the high frequency regime (noise) were identified, then the spatial resolutions were calculated as half of the reciprocal inverse of the spatial frequencies, which corresponded to (2.6 ± 0.1) μm for H&E sections, (14.2 ± 0.5) μm for IPC and (21.0 ± 1.4) μm for GPC.

The value of δ is approximately proportional to the mass density^24^. To measure the density resolution of GPC-CT, the standard deviation and the mean value of δ of the polypropylene container in GPC were calculated, which corresponded to 9.5 × 10^−9^ and 5.5 × 10^−7^ respectively. The mass density of the polypropylene was 910 mg/cm^3^, so that the estimated mass density resolution in GPC-CT was 15.7 mg/cm^3^. Similarly, the corresponding mass density resolution in IPC-CT was 25.5 mg/cm^3^.

**Figure 4:**
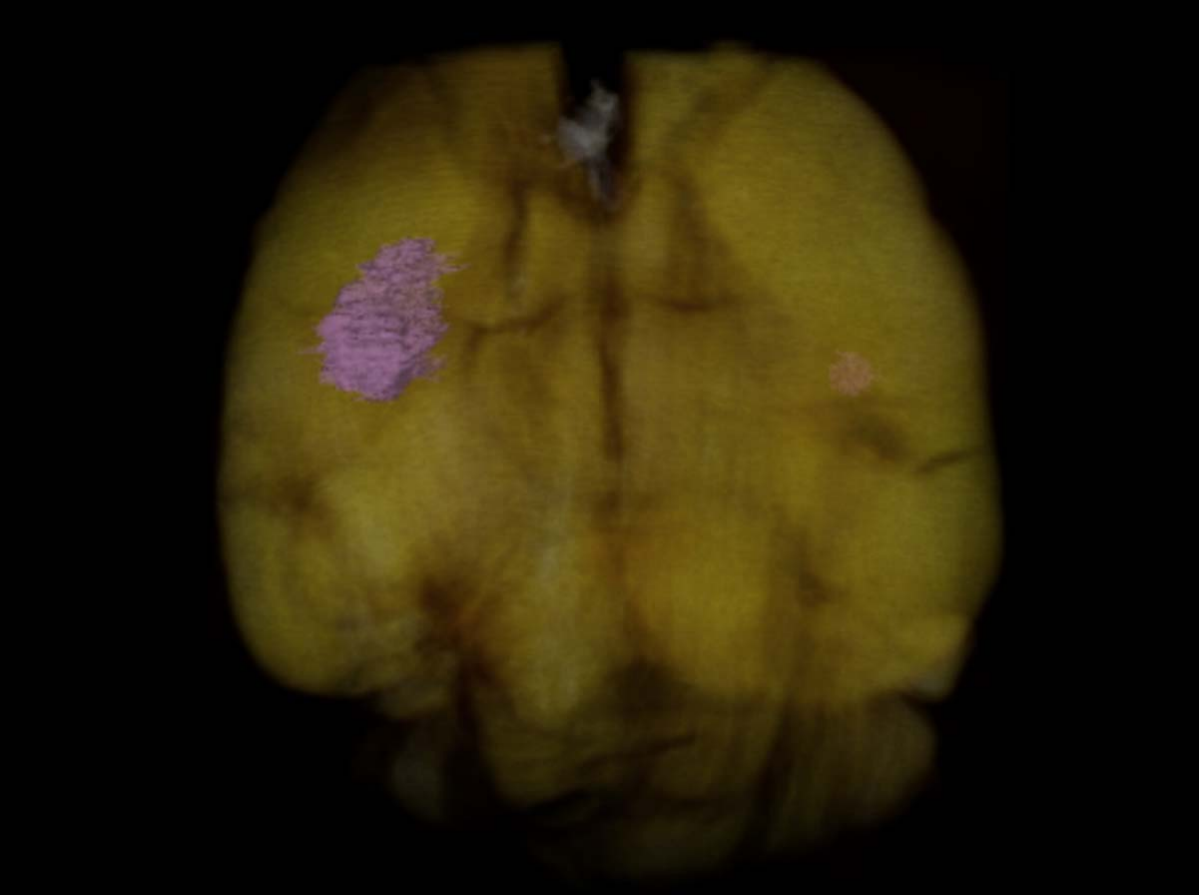
The reconstructed mouse brain structure by GPC tomography. Breast cancer metastatic foci in motor cortex and hippocampus are marked in pink.

The full BMs were visible after the GPC-CT tomogram was reconstructed in three dimensions (see Fig. 4 and Supplementary Video S1).

## Discussion

The phase-contrast imaging method has previously been used for analyzing brain micro-structure^23^ and diagnosing brain benign diseases^25^. However, few existing studies have used the phase-contrast imaging (PCI) method to study brain tumors. One previous study used PCI to image glioma in rat brain under contrast agent^26^. Hall *et al.*, tag C6 glioma cells with gold nanoparticles to increase their electron density and then implanted the tagged cells in the brains of Wistar rats^26^. The tumor with average size about 150 μm was visualized at Italy's Elettra synchrotron and the results were consistent with tissue histology. However, the implanted cells that were not loaded with gold nanoparticles were not detected. In our study, we did not use a contrast agent to increase the electron density in the brain metastatic foci. Instead, metastases were detected by both IPC-CT and GPC-CT based on phase-contrast only.

In another study by Pfeiffer *et al.*, GPC was used to visualize brain tumor without contrast agent^16^. The experimental animal which they used was a Fisher 344 rat, and the tumor cells, 9 L gliosarcoma, were directly injected into the brain. Eventually, the brain tumor's diameter increased to approximately 5 mm, at which point it was detected. Our experiment is different from this previous study in the way that we used a small animal model of six-week-old female WT BALB/C mouse, which was much smaller than Fisher 344b rat. BALB/C mice injected with human breast cancer cells from tail vein form brain metastases, which is consistent with a human tumor metastasis pattern. This is different from the procedure of direct injection of cells into brain which might destroy the brain structure. Additionally, the diameters of the metastases visualized by IPC-CT and GPC-CT in the brain were much smaller. In our experiment, the size of the motor cortex micrometastasis is approximately 660 × 580 × 770 μm^3^, and the size of the hippocampus micrometastasis is approximately 280 × 200 × 200 μm^3^. In particular, because the metastases had developed for 12 weeks, certain portions of the metastases borderline displayed the cancer's most important invasion characteristics, i.e., a crab's feet-like infiltration, whose morphological features are very clear in our GPC image. And the images from both IPC and GPC studies are consistent with histological examination similar to previous studies^27^,^28^.

In the current study, we also make the morphological comparison and quantitative analysis to discuss the feasibility and difference of GPC and IPC in the detection of the micrometastasis in brain. Different X-ray phase-contrast imaging methods have different characteristics. In general, GPC can provide a quantitatively correct reconstruction of the δ value for multi-material samples, which could be used to study and give high-resolution images on complex biological samples and give high-resolution images to visualize, e.g., human carotid arteries^29^, mouse body^30^, and human breast cancer tissues^31^. This is normally difficult for single-distance IPC. Additionally, compared with single-distance IPC, GPC scan can simultaneously yield phase-contrast, absorption-contrast, and dark-field images to provide more information of the samples^32^. However, the GPC experimental setup is much more complex than that of IPC. And the complex imaging acquisition scheme and data reconstruction procedure will also take much more time in both the experiment and the subsequent data analysis^33^.

Other than the apparent differences, the slight different contrast mechanisms of both phase-contrast imaging methods can lead to discernable difference in real imaging studies. Some studies have already been carried out to evaluate the capabilities of different phase-contrast imaging methods for the study of biological specimens^27^,^31^,^33^,^34^. In our experiment, both IPC and GPC could identify the micrometastasis in brain based on morphological and δ value variation from the peripheral normal tissues, and the CNR of both methods is quite similar for a big metastasis. However, for a small metastasis, it was much easier to be visualized in GPC than in IPC since the CNR of GPC was higher than that of IPC, which makes the abnormal structures more recognizable despite the fact the IPC-CT setup has higher spatial resolution. It suggests that the density resolution plays a more important role in the detection of micrometastasis in our experiment.

It should be noted that the current experiment has not been optimized for dose minimization. And the accumulated doses in our study as listed in Table 1 are on the order of hundreds Gy for both IPC-CT and GPC-CT scan. The dosage is much higher than the average lethal dose for *in vivo* imaging of a living rat. Some improvements can be applied to reduce the dose in future research, e.g., we could improve the detective efficiency of the image acquisition system to decrease the exposure time especially in IPC-CT scan, additionally, advanced acquisition scheme can be used instead of the traditional phase-stepping method to reduce the stepping number in single projection in GPC-CT scans^35^,^36^.

In this study, we used only the first-order Talbot distance in the GPC-CT setup because of the source size coherence limitation. By improving the coherence or introducing another splitter grating in front of the sample, a higher order Talbot distance can be accessed, which will significantly improve the method's density sensitivity in differentiating abnormal structures. Additionally, due to the time limitation of current experiment, our specimen was dehydrated before the imaging study. This made the specimen differ from the original morphologic features, which led to some imaging artifacts in the cingulated cortex region in IPC. We note that sample fixed in formalin^16^,^19^,^20^, especially sample prepared in phosphate buffered saline(PBS)^37^ would be more close to the real *in vivo* condition compared to our current preparation. Overall, in future research, we will focus on optimizing the GPC imaging technique with regards to the hardware set-up and the image acquisition scheme processing to detect mouse brain micrometastases close to *in vivo* condition, as this will more closely mimic its clinical applications.

## Methods

### Ethics Statement

This study was performed in strict accordance with the recommendations in the Guide for the Care and Use of Laboratory Animals of Fudan University. The protocol was approved by the Committee on the Ethics of Animal Experiments of Fudan University (Permit Number, SYXK 2012-0001). The surgeries were performed under pentobarbital sodium deep anesthesia, and all efforts were made to minimize suffering.

### Animal model and brain sample preparation

Pathogen-free six-week-old female WT BALB/C mice were purchased from the Shanghai Medical College of Fudan University and housed in standard animal cages under specific pathogen-free conditions in the college's animal facility. The mice were maintained in accordance with the Institutional Animal Care Guidelines and fed a regular basal diet and tap water ad libitum. We used 2 × 10^5^ MDA-MB-231 high metastasis (MDA-MB-231 HM) cells, which we previously constructed^38^; the cells were diluted in 0.1 ml of 0.9% normal saline and injected into the mouse tail vein. After 12 weeks, the mice were deeply anesthetized and euthanized with an overdose of 10% pentobarbital sodium. The brain was fully excised and fixed with 4% paraformaldehyde in PBS (pH 7.0) over 48 h at 4°C; graded ethanol was then used to dehydrate the samples. The samples were dried completely at 37°C for 24 h. Then the sample was imaged first with IPC-CT and then with GPC-CT.

### Phase-contrast imaging with SR X-rays

The synchrotron experiments were performed at the X-ray imaging and biomedical application beamline (BL13W1) at the Shanghai Synchrotron Radiation Facility (SSRF).

At the beamline, X-rays are emitted from a wiggler source with a wide energy spectrum (8–70 KeV). The X-ray source is 400 (H) μm × 24 (V) μm and located approximately 30 m away from the sample stage. The monochromatic X-ray beam from a double-crystal Si (111) monochromator had a corresponding beam size of 40 (H) mm × 3.5 (V) mm at the sample stage. Both IPC-CT and GPC-CT were performed on the brain samples.

### Image acquisition and processing with the IPC imaging method

The IPC set-up has a simple geometry, including a scintillator, a 45° mirror and an optically-coupled detector. The sample was placed 25 cm in front of the scintillator. The detector is a CCD camera (PCO2000, PCO, Germany) with pixel size of 7.4 μm. And it was coupled with a 2× microscope objective. The effective pixel size was 3.7 μm. An X-ray energy of 16 keV was used for the CT scan. We collected images at 1,080 projection angles for the 180°-rotation scan. At each projection, the exposure time was 2 s with an exposure dose about 190 mGy.

We used the Paganin algorithm for single-distance phase retrieval on each projection image^39^. Then the tomographic reconstruction was carried out by the standand filtered back projection (FBP) algorithm.

### Image acquisition and processing with the GPC imaging method

In addition to the scintillator, the 45° mirror, and the optically-coupled detector, the GPC set-up also included one phase grating (G1) and one absorption grating (G2). G1 consists of nickel stripes (3.5 μm height) on the silicon substrate with a 2.4 μm period and introduces a π/2 phase shift. G2 was consists gold stripes (50 μm height) on the silicon substrate with the same period as G1. Both gratings were manufactured by Mircoworks, Inc. (Karlsruhe, Germany). We used a pco.edge sCMOS detector (PCO, Germany) with pixel size of 6.5 μm. It was coupled with a 1× camera lens. The detector was used mainly for its fast acquisition speed while maintaining reasonable signal-to-noise ratio, as more images have to be collected in GPC-CT compared with IPC-CT. The first-order Talbot distance was used at the X-ray energy of 20 keV, which corresponds to a 4.64 cm distance between G1 and G2. The detector was placed immediately after G2. The sample was placed approximately 10 cm before G1 because of the geometric constraints.

We used the conventional phase-stepping method in our imaging process. G1 was scanned for six steps, and six images were recorded at each projection angle with a step distance of 0.4 μm, which was controlled by a highly sensitive step motor (Kohzu, Japan). The exposure time was 100 ms and single exposure dose was 25 mGy. We collected images at 720 projection angles for a 180° rotation scan and reference images at 120 projection angles during the scan for image normalization.

We used a fast Fourier transform method to extract the phase informations^32^ and obtain the 3D distribution of δ using the Hilbert-filter-based filtered back projection (FBP) algorithm^40^.

### Histology

After the imaging study was performed, the sample was paraffin embedded using a standard technique; 4-μm-thick sections were prepared using a rotary microtome (Leica RM2235) and placed on glass slides. The brain was serially sectioned, and each slice was stained with hematoxylin and eosin (HE). One pathologist confirmed the presence of cancer cells in the brain tissue.

### Registration

Manual and computationally automatic registrations were combined in our registration process. A set of feature points with high local density were obtained using the SURF algorithm (open source code by D. Kroon^41^) following the original work by N. Chicherova *et al*^42^. To reduce the iterations in the plane fitting and avoid the misalignment, some prior alignment information from the manual registration process was introduced. The feature points within a distance of 20 pixels to the manually selected slices were used to fit the most representative slices to the H&E sections.

## Author Contributions

S.H., Y.Y.C. and J.W. conducted the experiments, performed the IPC tomography scanning and wrote the manuscript, B.Q.K., Y.X.C., J.Q.S. and Y.J.W. performed the GPC tomography scanning, analysed the data, image reconstruction and wrote the manuscript. Y.X. analyzed the IPC data. Z.M.S., T.Q.X. and J.Z. gave scientific advice. W.L.C. confirmed the brain pathological changes. H.G. and Y.N.F. helped to perform the synchrotron experiment. X.H. helped to perform the animal experiment.

## Supplementary Material

Supplementary Information Supplementary Information

Supplementary Information The reconstructed 3D structure of the mouse brain by GPC tomography.

## Figures and Tables

**Table 1:** Relevant imaging conditions of two samples

	IPC	GPC
**Motor cortex micrometastasis**	6.17 ± 0.12	5.05 ± 0.18
**Hippocampus micrometastasis**	1.02 ± 0.09	2.72 ± 0.19
**Dose (Single projection)**	190 mGv	25 mGv
**Accumulated Dose**	205 Gv	108 Gv
