# Supplementary material for: In-line phase-contrast and grating-based phase-contrast synchrotron imaging study of brain micrometastasis of breast cancer
Source: Sci Rep. 2015 Mar 30;5:9418. doi: 10.1038/srep09418 (PMC4377630; doi:10.1038/srep09418)
Supplement: Supplementary Information [file srep09418-s1.pdf]

In-line phase-contrast and grating-based phase-contrast synchrotron imaging study of brain micrometastasis of breast cancer

Sheng Huang[a,1], Binquan Kou[b,1], Yayun Chi[a,1], Yan Xi[c,d], Yixin Cao[b], Wenli Cui[e,f], Xin Hu[a], Zhimin Shao[a], Han Guo[g], Yanan Fu[g], Tiqiao Xiao[g], Jianqi Sun[c], Jun Zhao[c,d], Yujie Wang[b,*], Jiong Wu[a,*]

[a] Department of Breast Surgery, Breast Cancer Institute, Shanghai Cancer Center, Department of Oncology, Shanghai Medical College, Fudan University, Shanghai, People's Republic of China

[b] Department of Physics and Astronomy, Shanghai Jiao Tong University, Shanghai, People's Republic of China

[c] School of Biomedical Engineering, Shanghai Jiao Tong University, Shanghai, People's Republic of China

[d] Med-X Research Institute, Shanghai Jiao Tong University, Shanghai, People's Republic of China

[e] Department of pathology, Fudan University, Shanghai Cancer Center, Shanghai, People's Republic of China

[f] Department of pathology, First Affiliated Hospital Xinjiang Medical University, Urumqi, Xinjiang Uygur Autonomous Region, People's Republic of China

[g] Shanghai Institute of Applied Physics, Chinese Academy of Sciences, Shanghai, People's Republic of China

Supplementary Information

Video Legend

Supplementary Video S1. The reconstructed 3D structure of the mouse brain by GPC tomography. The breast cancer metastatic foci in motor cortex and hippocampus are marked in pink.
